# Synergistic Interactions between the NS3_hel_ and E Proteins Contribute to the Virulence of Dengue Virus Type 1

**DOI:** 10.1371/journal.pntd.0001624

**Published:** 2012-04-17

**Authors:** Luana de Borba, Daisy M. Strottmann, Lucia de Noronha, Peter W. Mason, Claudia N. Duarte dos Santos

**Affiliations:** 1 Laboratório de Virologia Molecular, Instituto Carlos Chagas (ICC–FIOCRUZ/PR), Curitiba, Paraná, Brazil; 2 Laboratório de Patologia Experimental, Pontifícia Universidade Católica do Paraná (PUC/PR), Curitiba, Paraná, Brazil; 3 Department of Pathology, University of Texas Medical Branch (UTMB), Galveston, Texas, United States of America; University of North Carolina at Chapel Hill, United States of America

## Abstract

**Background:**

Dengue includes a broad range of symptoms, ranging from fever to hemorrhagic fever and may occasionally have alternative clinical presentations. Many possible viral genetic determinants of the intrinsic virulence of dengue virus (DENV) in the host have been identified, but no conclusive evidence of a correlation between viral genotype and virus transmissibility and pathogenicity has been obtained.

**Methodology/Principal Findings:**

We used reverse genetics techniques to engineer DENV-1 viruses with subsets of mutations found in two different neuroadapted derivatives. The mutations were inserted into an infectious clone of DENV-1 not adapted to mice. The replication and viral production capacity of the recombinant viruses were assessed *in vitro* and *in vivo*. The results demonstrated that paired mutations in the envelope protein (E) and in the helicase domain of the NS3 (NS3_hel_) protein had a synergistic effect enhancing viral fitness in human and mosquito derived cell lines. E mutations alone generated no detectable virulence in the mouse model; however, the combination of these mutations with NS3_hel_ mutations, which were mildly virulent on their own, resulted in a highly neurovirulent phenotype.

**Conclusions/Significance:**

The generation of recombinant viruses carrying specific E and NS3_hel_ proteins mutations increased viral fitness both *in vitro* and *in vivo* by increasing RNA synthesis and viral load (these changes being positively correlated with central nervous system damage), the strength of the immune response and animal mortality. The introduction of only pairs of amino acid substitutions into the genome of a non-mouse adapted DENV-1 strain was sufficient to alter viral fitness substantially. Given current limitations to our understanding of the molecular basis of dengue neuropathogenesis, these results could contribute to the development of attenuated strains for use in vaccinations and provide insights into virus/host interactions and new information about the mechanisms of basic dengue biology.

## Author Summary

Dengue virus constitutes a significant public health problem in tropical regions of the world. Despite the high morbidity and mortality of this infection, no effective antiviral drugs or vaccines are available for the treatment or prevention of dengue infections. The profile of clinical signs associated with dengue infection has changed in recent years with an increase in the number of episodes displaying unusual signs. We use reverse genetics technology to engineer DENV-1 viruses with subsets of mutations previously identified in highly neurovirulent strains to provide insights into the molecular mechanisms underlying dengue neuropathogenesis. We found that single mutations affecting the E and NS3_hel_ proteins, introduced in a different genetic context, had a synergistic effect increasing DENV replication capacity in human and mosquito derived cells *in vitro*. We also demonstrated correlations between the presence of these mutations and viral replication efficiency, viral loads, the induction of innate immune response genes and pathogenesis in a mouse model. These results should improve our understanding of the DENV-host cell interaction and contribute to the development of effective antiviral strategies.

## Introduction

Dengue virus (DENV) is an arthropod-borne flavivirus that belongs to the family *Flaviviridae*. The DENV genome is a 11 kb single-stranded RNA molecule of positive polarity that encodes a single open read frame (ORF), which is flanked by two untranslated regions (5′ and 3′UTR) [1]–[2], which are involved in viral RNA replication and translation [3]–[6]. ORF translation generates a single polyprotein that is cleaved by host and virus-derived proteases to produce three structural (C, prM and E) and seven non-structural proteins (NS1, NS2A, NS2B, NS3, NS4A, NS4B and NS5) [1].

The four serotypes of DENV (DENV-1 to DENV-4) are transmitted to humans by the mosquito vector *Aedes aegypti*. Dengue disease is endemic to subtropical and tropical countries, and the World Health Organization (WHO) estimates that 50 to 100 million individuals become infected annually. DENV infection results in a spectrum of illnesses, ranging from a flu-like disease (dengue fever, DF) to more severe and potentially fatal, dengue hemorrhagic fever (DHF) and dengue shock syndrome (DSS) [7]–[8]. Epidemics with high frequencies of DHF/DSS are spreading throughout South America and unusual clinical presentations such as encephalitis, hepatitis and other visceral signs are becoming more frequent [9]–[12]. There are currently no vaccines or specific licensed antiviral drugs for prevention or treatment of dengue [13]–[15].

Despite major advances in DENV biology, many aspects of dengue pathogenesis remain largely unknown. Animal models reproducing some of the salient features of dengue disease have been used to investigate the underlying pathogenesis mechanisms. Multiple lines of evidence indicate that immunopathological mechanisms play an important role in the development of DHF/DSS [7], [16]. The prevalence of DHF is higher in patients experiencing secondary infection with a heterotypic dengue virus serotype, leading to the suggestion that severe disease may result from antibody dependent enhancement (ADE) [17]–[21]. However, severe disease is often observed after primary infections, indicating a role for individual strains of DENV, in addition to host factors related to previous infection in the development of severe dengue disease [22]–[25]. Disease severity is thus probably determined by the interplay of viral and host factors. Several mouse models of dengue disease have been described, but even those that faithfully reproduce some features of human disease, present limitations because they are based on the use of mouse-adapted viruses or genetically modified animals. Nevertheless, these models have provided insights into DENV pathogenesis.

Many studies have shown that mutations affecting the E protein, which covers the flavivirus surface, can alter flavivirus virulence. The E protein, a glycosylated dimeric membrane protein [26], interacts with receptors on the host cell surface [27]–[28], mediating virus binding and fusion to the host cell membrane [29]–[31] and conferring protective immune responses by eliciting antibody production [32]–[33].

Prestwood and coworkers [34] described a DENV-2 isolate that had been obtained by passing a clinical isolate in mosquitoes and mice, and that caused severe disease in AG129 mice. By reverse genetic techniques, they identified two mutations affecting the E protein (E_124_ and E_128_) as responsible for an increase in virulence. The recombinant virus had a low affinity for heparin sulfate, reducing its binding to cells and increasing its half-life in the serum. This would potentially allow a larger number of viral particles to infect the visceral tissues thereby increasing disease severity in this mouse model.

NS3 protein is one of the most highly conserved proteins in flaviviruses. This multifunctional protein has at least three different activities [35]. It has a serine protease domain that catalyzes the cleavage of several viral proteins, an RNA helicase domain, and an RNA triphosphatase domain, which promotes dephosphorylation of the 5′UTR region during capping activities [36]–[46]. In the course of human dengue infection, NS3 is a common target of T cells [47].

The helicase domain of NS3 (NS3_hel_), together with NS5, an RNA-dependent RNA polymerase, participates in viral RNA replication and it is essential for genome propagation. It has been demonstrated that the interaction between DENV NS3_hel_ and NS4B triggers the dissociation of the helicase from single-stranded RNA thereby modulating viral replication. The enzymatic activities and role of NS3 proteins in viral replication and polyprotein processing have been studied for several members of the *Flaviviridae* family [48]–[50], but only a few studies have identified point mutations in NS3 modulating viral pathogenesis.

We previously described neurovirulent variants of DENV-1 that were generated by adapting viruses to cause lethal neurological disease in mice [51]–[52]. Comparisons of the sequences of parental and mouse-adapted strains identified mutations affecting positions 402 and 405 of E protein, and in the helicase domain of the non-structural protein NS3 (positions 209, 435 and 480), as potentially responsible for this neurovirulent phenotype [52]–[53]. We evaluated the viral molecular determinants putatively identified as contributing to DENV pathogenesis in a mouse model, by introducing each mutation, individually or in combination, into a non-neurovirulent infectious cDNA clone of DENV-1 and recovering genetically defined DENV-1 strains which were then used to determine the effect of these mutations *in vitro* and *in vivo*. These results build on previous demonstrations that multiple mutations in different regions of the genomes of dengue and other flaviviruses cooperate in the modulation of pathogenesis [54]–[56].

## Methods

### Ethics statement

Animal experiments were approved by the ethics committee for animal experimentation of the Federal University of Parana (CEP/UFPR 23075-0429663/2007-97). The procedures using animals in this research project are specified in accordance with the ethical principles established by the Brazilian College of Animal Experimentation (COBEA) and requirements established in “Guide for the Care and Use of Experimental Animals (Canadian Council on Animal Care)”.

### Cell cultures

*Aedes albopictus* cells (C6/36) were grown at 28°C in Leibovitz L-15 medium (Gibco/Invitrogen, Grand Island, NY, USA) supplemented with 0.26% Tryptose (Sigma-Aldrich, St. Louis, MO, USA), 25 µg/mL gentamicin (Gibco/Invitrogen, Grand Island, NY, USA) and 5% fetal bovine serum (FBS) (Gibco/Invitrogen, Grand Island, NY, USA). Human hepatoma cells (Huh7.5) were grown in 37°C, under an atmosphere containing 5% CO_2_, in Dulbecco's Modified Eagle Medium: Nutrient Mixture F-12 (DMEM/F12) (Gibco/Invitrogen, Grand Island, NY, USA) supplemented with 25 µg/mL gentamicin and 10% FBS. Neuroblastoma cells (Neuro-2a) were grown in 37°C, under an atmosphere containing 5% CO_2_, in Dulbecco's Modified Eagle Medium (DMEM) (Gibco/Invitrogen, Grand Island, NY, USA) supplemented with 1x non essential amino acids (Gibco/Invitrogen, Grand Island, NY, USA), 25 µg/mL gentamicin and 5% FBS.

### Infectious cDNA clones

All clones were constructed using the backbone of the infectious genome-encoding plasmid pBACDV1 [57] (a bacterial artificial chromosome plasmid – pBAC). The pBACDV1 consists of the full-length cDNA of strain BR/90 (differing from the sequence deposited in GenBank (AF226685.2) by only 11 nucleotides, and none of which results in an amino-acid substitution), a T7 RNA polymerase promoter sequence with a single-non-genomic G residue introduced immediately upstream from the first nucleotide of the 5′UTR (to ensure high levels of synthetic transcript production), and a hepatitis delta virus ribozyme sequence (HDV-RZ) followed by a unique restriction endonuclease site just after the last nucleotide of the 3′UTR, (to facilitate the production of templates for RNA synthesis) [57].

### Construction of recombinant DENV clones

To construct the recombinant cDNA clones containing the mutations identified in the neurovirulent DENV-1 strains, overlapping polymerase chain reaction (PCR) amplifications to generate cDNA molecules containing specific mutations, except for the NS3_435_ mutation, which was located very close to a naturally occurring restriction endonuclease site, making it possible to incorporate this mutation into the DENV-1 cDNA through the use of a single mutated oligonucleotide. All amplifications were carried out with the high fidelity enzymes of the TripleMaster System (Eppendorf, Westbury, NY, USA) or LongRange PCR (Qiagen, Valencia, CA, USA), following the manufacturer's protocols. In some cases, the fragments containing the desired mutations were initially inserted into the pGEM-T Easy Vector System (Promega, Madison, WI, USA), in accordance with manufacturer's instructions. The desired infectious cDNAs were reconstructed by using the corresponding fragments obtained either directly from the PCR amplicon, or from the pGEM-T clone to replace the parental fragments in the DENV-1 infectious genome in pBACDV1.

**Figure 1:**
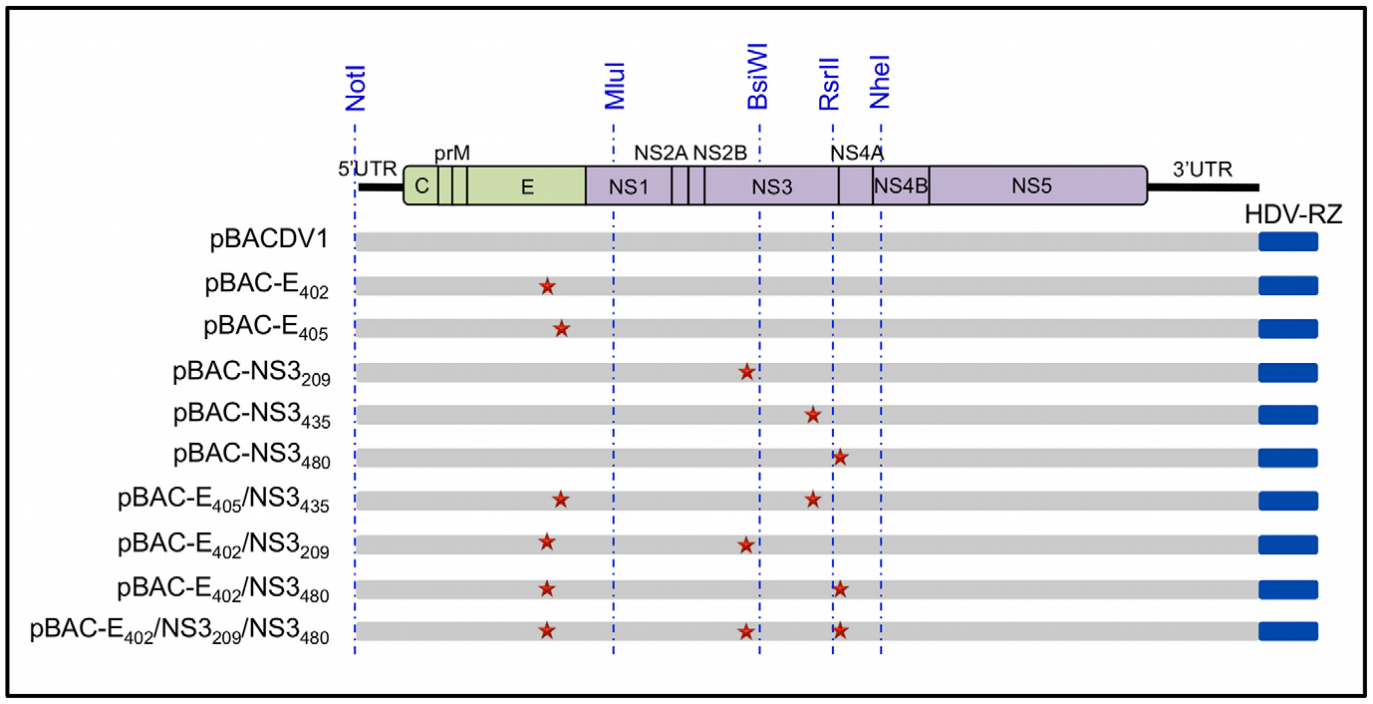
Line diagrams representing the structure of recombinant genomes and the position of key viral elements. Dotted lines show the positions of the restriction endonuclease sites used to insert fragments containing mutations (represented by stars) into pBACDV1.

The fragments replaced for each mutation were: a NotI/MluI fragment for the E mutations (E_402_ and E_405_), a BsiWI/RsrII fragment for the NS3_435_ mutation, a MluI/BsiWI fragment for the NS3_209_ mutation, and a BsiWI/NheI fragment for the NS3_480_ mutation (Figure 1). The clones with individual mutations were named: pBAC-E_402_, pBAC-E_405_, pBAC-NS3_209_, pBAC-NS3_435_ and pBAC-NS3_480_, respectively. Finally, for the construction of the double and triple mutants, we combined the E-mutation with the NS3-mutation found in two independent neuroadapted strains (Table 1), generating the clones pBAC-E_405_/NS3_435_, pBAC-E_402_/NS3_209_, pBAC-E_402_/NS3_480_ and pBAC-E_402_/NS3_209_/NS3_480_. Each construct was confirmed by sub mitting the replaced fragment for sequencing, at the Macrogen Sequencing Service (Seoul, South Korea).

**Table 1:** Summary of amino-acid sequence differences between the vBACDV1 and the panel of DENV infectious cDNA clones containing the selected subset of substitutions in the E and/or NS3 proteins.

Coding region	Codon position^a^	FGA/89^b^	FGA/NA d1d^c^	FGA/NA P6^d^	vBAC DV1^e^	vBAC-E_402_	vBAC-E_405_	vBAC-NS3_209_	vBAC-NS3_435_	vBAC-NS3_480_	vBAC-E_405_/NS3_435_	vBAC-E_402_/NS3_209_	vBAC-E_402_/NS3_480_	vBAC-E_402_/NS3_209_/NS3_480_
**E**	**402**	F	F	**L** *	F	**L** *	F	F	F	F	F	**L** *	**L** *	**L** *
**E**	**405**	T	**I** *	T	T	T	**I** *	T	T	T	**I** *	T	T	**T**
**NS3**	**209**	V	V	**I** *	V	V	V	**I** *	V	V	V	**I** *	V	**I** *
**NS3**	**435**	L	**S** *	L	L	L	L	L	**S** *	L	**S** *	L	L	**L**
**NS3**	**480**	L	L	**S** *	L	L	L	L	L	**S** *	L	L	**S** *	**S** *

a Position of codon change within the individual protein-encoding region.

b Parental strain from which the neuroadapted strains were derived (AF226686.2) [45]–[46].

c Neuroadapted strain (AF226686.1).

d Neuroadapted strain (EF122231.1).

e vBACDV1 differs from the original BR/90 GenBank-deposited sequence (AF226685.2) by only 11 synonymous nucleotide substitutions; both BR/90 and vBACDV1 are identical to the parental virus used for previous neuroadaptation studies (strain FGA/89; [45]–[46]) for the 5 codons shown in this table (see Table S1).

*: Mutations studied.

### RNA transcription and transfection

Infectious DENV RNAs were generated by linearizing the recombinant pBAC DNAs in an overnight digestion at 25°C with SwaI (New England Biolabs, Ipswich, MA, USA), purifying the products by phenol extraction and ethanol precipitation and transcribing them *in vitro* with T7 RNA polymerase in the presence of an 7 mG(ppp)G RNA cap analog (Biolabs, Ipswich, MA, USA) with the T7 MEGAScript Transcription System (Ambion, Austin, TX, USA). Eight individual wells of C6/36 cells cultured at 28°C were transfected with RNA transcripts in the presence of Lipofectin (Invitrogen, Carlsbad, CA, USA). Supernatant samples were harvested in duplicate at 48, 72, 96 and 120 hours after transfection, and used for viral titration. The time points with the highest titers were used for subsequent viral amplification.

### Titration

Viral titers were determined by the focus-forming unit technique in C6/36 cells (ffu_C6/36_), as previously described [58]. Foci were immunostained with purified supernatants of the *Flavivirus* group-specific mouse monoclonal antibody 4G2, and the bound antibodies were then decorated with goat anti-mouse immunoglobulin conjugated to alkaline phosphatase (Promega, Madison, WI, USA), which was detected by adding a solution of NBT (nitro-blue tetrazolium chloride) and BCIP (5-bromo-4-chloro-3′-indolyphosphate p-toluidine salt) (Promega, Madison, WI, USA) as a substrate.

### Viral amplification and purification

To increase viral titers and generate working stocks, two rounds of infection were performed with each of the recovered virus, using the time point with highest titer in RNA transfection experiments *in vitro*. The first round of amplification was performed in T25 flasks (TPP, Trasadingen, Switzerland) of C6/36 cells (5×10^5^ cells/flask) at a multiplicity of infection (MOI) of 0.01. The cell cultures were incubated at 28°C until cytopathogenic effects were observed or, in some cases, infection was confirmed by routine indirect immunofluorescence assays, six days after infection (data not shown). Virus yields for each sample were determined by titration, as described above. The second round of amplification was performed in T300 flasks (TPP, Trasadingen, Switzerland) (2×10^7^ C6/36 cells/flask), under the same conditions as described above. Recombinant viruses were purified from the products of this second amplification by centrifugation on a sucrose gradient, as previously described [59]. A mock-infected control preparation was prepared from non-infected C6/36 cells by the same protocol.

### Complete genome sequencing

Viral RNA was purified from sucrose gradient stocks, using the QIAamp Viral RNA Mini Kit (Qiagen, Valencia, CA, USA). The resulting RNA was reverse transcribed with the Improm-II Reverse Transcriptase (Promega, Madison, WI, USA) in the presence of random primers (100 pmol/µL – Invitrogen, Carlsbad, CA, USA) and the entire genome was amplified by PCR for nucleotide sequencing, which was carried out by the Macrogen Sequencing Service (Seoul, South Korea).

### *In vitro* kinetics analysis

Huh7.5 (4×10^5^ cells/well) and C6/36 (2×10^5^ cells/well) cells were infected in 24-multiwell plates (TPP, Trasadingen, Switzerland) with mock and recombinant viruses vBACDV1, vBAC-E_402_, vBAC-E_405_, vBAC-NS3_209_, vBAC-NS3_435_, vBAC-NS3_480_, vBAC-E_405_/NS3_435_, vBAC-E_402_/NS3_209_, vBAC-E_402_/NS3_480_ and vBAC-E_402_/NS3_209_/NS3_480_. A MOI of 5 was used to infect Huh7.5 cells by incubation for 1 h at 37°C under an atmosphere containing 5% CO_2_, and a MOI of 1 was used to infect C6/36 cells by incubation for 1 h at 28°C. Cells were recovered at 24, 48, and 72 hours post infection (hpi). The number of cells infected was determined by flow cytometry, according to previously described protocols [61]. Cells were analyzed with a FACS Canto II system (Becton & Dickinson, San Jose, CA). FACS data were analyzed with FlowJo 2.2.8 software.

### Cell binding assays

To determine the binding affinity of the recombinant viruses for Neuro-2a cells, Amicon (Millipore, Billerica, MA, USA) concentrated recombinant vBACDV1, vBAC-E_402_, vBAC-E_405_ viruses and a mock-infected control were incubated with 2×10^5^ Neuro-2a cells at MOI of 100 for 1 h at 4C°. The cells were then washed three times with ice-cold PBS to remove unbound virus. They were lysed and viral RNA was extracted with the QIAamp Viral RNA mini Kit (Qiagen, Valencia, CA, USA), according to the manufacturer's protocols. The number of bound genome-containing particles per cell was then determined by RT/qPCR in three independent experiments, as previously described [60]. The murine gene encoding GAPDH was also included as a housekeeping gene in all analysis, for data normalization [62].

### Mouse studies

A 50% lethal dose (LD_50_) assay was performed with virus recovered from the pBACDV1 clone (vBACDV1), to determine the optimum dose of recombinant viruses for the inoculation of mice. Individual litters of two-day-old *Swiss* mice were inoculated via intracerebral (i.c.) route with four ten-fold dilutions (corresponding to 100,000 ffu_C6/36_ to 100 ffu_C6/36_) of purified vBACDV1 virus or one dilution of purified mock-infected C6/36 culture fluid (equivalent to the highest tested concentration of vBACDV1). Animals were monitored for 21 days. We found that the LD_50_ was equivalent to 56,234 ffu_C6/36_ of vBACDV1. For comparative studies, 562 ffu_C6/36_ (corresponding to 10^−2^ LD_50_) aliquots of each of the recombinant viruses were compared side-by-side through the i.c. inoculation of three individual litters of two-day-old mice, replicating the methods originally described for DENV-1 neurovirulence in *Swiss* mice [51]. The animals were observed for 21 days to evaluate the morbidity and mortality. Eight days post infection (dpi), three animals were randomly selected from each litter, euthanized and their brains were harvested and pooled for the quantification of virus replication and gene induction. Ten dpi, one animal per group was euthanized, its brain was collected and fixed in a 10% buffered formalin solution for histological analysis. In addition, mouse brain and spine cord tissues were individually collected at 6, 8 and 10 dpi of animals infected with mock, vBACDV1 and vBAC-E_402_/NS3_209_/NS3_480_ for RT/qPCR and virus titration analysis.

### Quantification of DENV RNA levels by quantitative RT-PCR (RT-qPCR)

Total RNA was isolated from 30 mg of pooled 8 dpi mouse brain tissues infected with each DENV or the mock, with the RNEasy Mini kit (Qiagen, Valencia, CA, USA), according to the manufacturer's protocol. For the quantification of viral RNA in the brain tissues by RT/qPCR, we subjected 2 µg of each RNA sample to amplification with 400 nM specific DENV-1 oligonucleotides and 300 nM specific DENV-1 probe, with the MultiScribe Enzyme Plus RNase Inhibitor and TaqMan Universal RT-PCR Master Mix (Applied Biosystems, Foster City, IA, USA) in an ABI PRISM 7500 Detection System (Applied Biosystems, Foster City, IA, USA) as previously described [60]. The mouse GAPDH housekeeping gene was included in all analysis for data normalization as previously described [52].

### Relative quantification of mRNA levels by quantitative PCR (qPCR)

The RNA isolated from DENV- and mock-infected mouse brain tissues (pooled from three individuals from each group, as described above) was used for the quantification of mRNA levels for seven genes (Irf1, Psmb8, Usp18, C1r, IFNα, IFNβ and CCL5) selected on the basis of a previous study by Bordignon and coworkers [63]. For this purpose, 4 µg of each RNA sample were reverse transcribed with ImProm-II Reverse Transcriptase (Promega, Madison, WI, USA) and oligo-dT primers (10 µM) according to the manufacturer's protocol. The resulting cDNAs were then diluted to a concentration of 2 ng/µl and used for amplification by qPCR, as previously described [63]. Melting curves were used to check product specificity. Levels of mRNA for each selected gene were recorded as gene mRNA/murGAPDH mRNA induced by dengue virus infection in the central nervous system (CNS) of mice.

### Statistical analysis

The qPCR data are reported as means ± standard deviation (SD) and were analyzed by one-way ANOVA with Bonferroni's or Dunn's correction for multiple comparisons. *In vitro* growth kinetics data are reported as means ± standard deviation (SD) and were analyzed using two-way ANOVA followed by a Bonferroni's test. The level of significance for the analyses was set at *p*≤0.05. Mortality data were analyzed by plotting Kaplan-Meier survival curves and carrying out Log-rank (Mantel-Cox) multiple comparison test. The analyses were performed with GraphPad Software (Prism 5 for Mac OS X – version 5.0c, San Diego, CA, USA).

## Results

For identification of putative viral determinants on the phenotype of neuroadapted DENV-1 strains, we constructed a panel of DENV cDNA infectious clones containing a subset of mutations affecting the E and NS3 proteins selected in two separate studies of the neuroadaptation of the FGA/89 strain to newborn mice [51]–[52]. The mutations were introduced into a DENV-1 infectious clone not adapted to mice (pBACDV1, derived from the DENV-1 prototype strain (BR/90) – [57]). Comparisons of the sequences of the infectious clone, the neuroadapted isolates and the parental strain used to generate the neuroadapted strains (FGA/89) (Table S1), led us to focus on mutations at positions 402 and 405 in E and 209, 435, and 480 in NS3 for the studies described here (Table 1).

**Figure 2:**
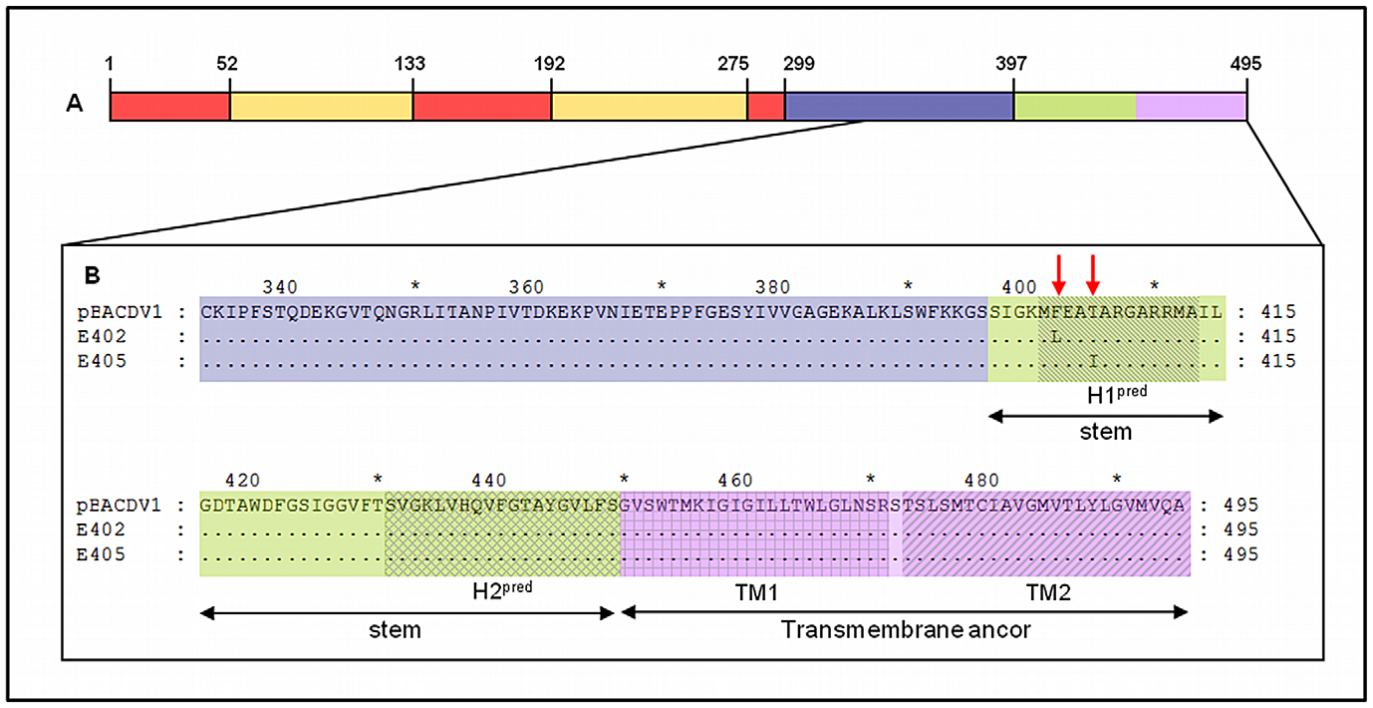
Location of Envelope mutations. (A) Representation of the full-length Envelope protein with domain I in red, domain II in yellow and domain III in blue. The stem region is represented in green and the transmembrane anchor in purple. (B) Detailed view of amino acids 333–495 showing the predicted regions H1^pred^ and H2^pred^ of stem, and domains TM1 and TM2 of the transmembrane anchor. The red arrows indicate the positions of mutations.

The mutations affecting E (E_402_ and E_405_) acquired during adaptation were found to be located outside the parts of the protein used for structural determinations by X-ray crystallography. Both these mutations lie within the first of two predicted α-helical structures H1^pred^ in the stem region of E just after the ectodomain [64] (Figure 2). This stem region seems to be involved in the formation of the E homotrimer, the interactions between E and prM, particle formation and intracellular retention [64]–[68].

**Figure 3:**
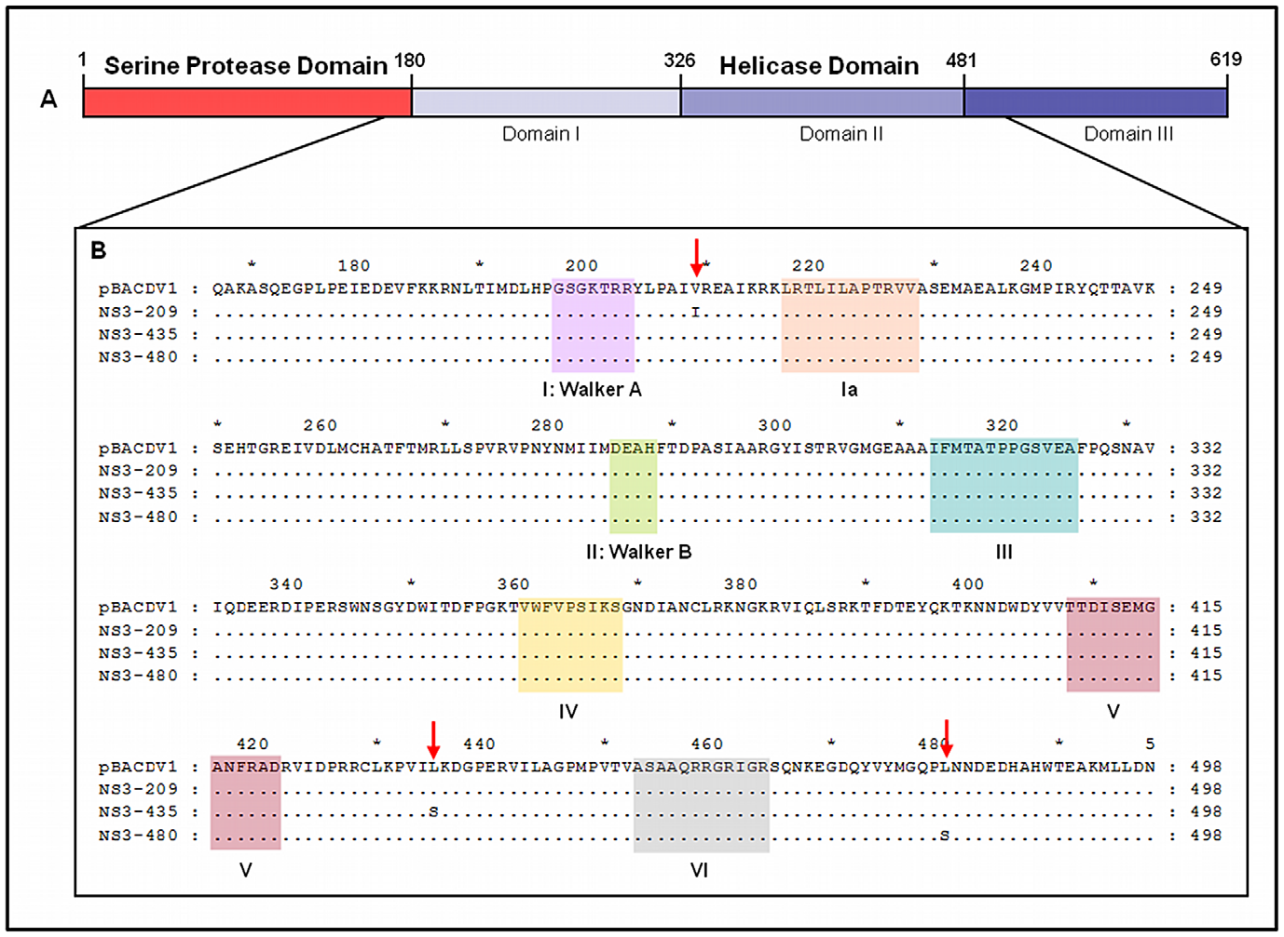
Location of NS3 mutations. (A) Representation of the full-length NS3 protein with the serine protease domain in red and the helicase domain in blue, with different shades of blue fused to represent the various subdomains. (B) Detailed view of amino acids 167–498, encompassing subdomains I and II of the helicase. Motifs conserved in helicase superfamily 2 are indicated in different colors and labeled. The red arrows indicate the positions of mutations.

The NS3 mutations acquired during neuroadaptation are located in the helicase domain, with the NS3_209_ mutation in subdomain I, and mutations NS3_435_ and NS3_480_ in subdomain II [45] (Figure 3). The helicase domain of the NS3 protein appears to be responsible for supporting the initiation of (−)ssRNA synthesis, through the unfolding of RNA secondary structures, providing access to the replication machinery [36], [69]–[70].

We investigated the effect of these mutations both individually and in combination on the *in vitro* and *in vivo* properties of DENV-1, by using infectious cDNAs harboring the mutations (Figure 1 and Table 1) as a source for *in vitro* RNA synthesis. The RNAs generated were then introduced into C6/36 cells for the recovery of viruses, which were amplified and purified as described in the Methods section. Analyses of the complete sequences of the genomes of all of the amplified viruses confirmed their identity with the pBACs used to generate them and showed that no adventitious mutations had been produced in the cloning steps or arisen during virus recovery and propagation.

**Figure 4:**
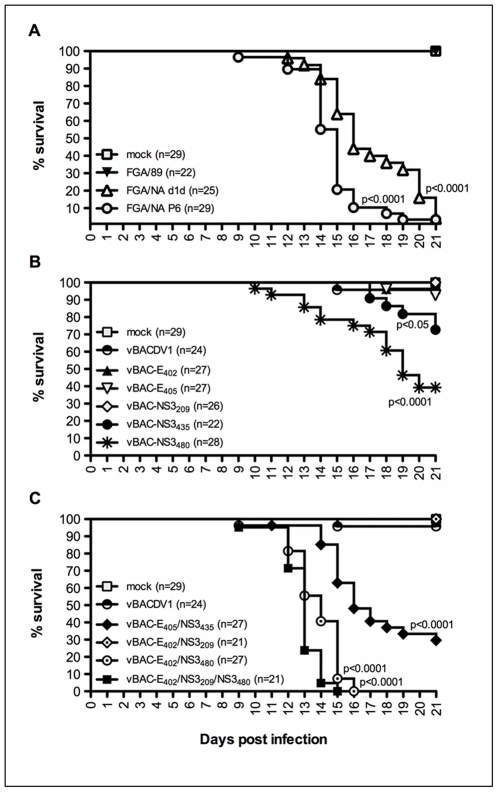
Newborn mice survival after i.c. inoculation with DENV-1 variants. (A) Comparison of mock, FGA/89 and neurovirulent strains, FGA/NA d1d and FGA/NA P6, (B) Comparison of mock, vBACDV1 and single-mutant recombinant viruses, (C) Comparison of mock, vBACDV1 and double- and triple-mutant recombinant viruses. Data from three independent experiments were pooled and plotted as Kaplan-Meier survival curves and then analyzed by log-rank (Mantel-Cox) multiple comparison tests, the *p* value for comparisons between FGA/89 and neuroadapted viruses, or vBACDV1 and the corresponding recombinant virus are indicated, and *n* is the total number of mice per group.

To evaluate the role of each mutation in the neurovirulent phenotype in a mouse model, purified recombinant viruses were inoculated i.c. in newborn *Swiss* mice. Three litters of mice, each containing 5 to 11 animals, were used. All inoculations were performed with a single dose of virus (562 ffu_C6/36_), corresponding to 1/100 LD_50_ for the parental cDNA clone-derived virus, vBACDV1 (see Methods, all viral genomes were resequenced before inoculation). The equivalent viral genomic RNA (GE) to FFU ratio (562 ffu_C6/36_) for each virus *inocula* was determined by RT/qPCR as previously described [61] to assure the comparability of viral infection doses (data not shown). Figure 4 shows the combined mortality data for three experiments. The animals inoculated with mock, FGA/89, vBACDV1, vBAC-E_402_, vBAC-E_405_, vBAC-NS3_209_ and vBAC-E_402_/NS3_209_ viruses survived forthe entire 21-day observation period. Mice in the groups inoculated with FGA/89, vBACDV1, vBAC-E_402_ and vBAC-E_402_/NS3_209_ behave normally throughout the observation period, whereas animals from the groups infected with vBAC-E_405_ and vBAC-NS3_209_ displayed mild signs of disease (Figure S1). However, all the animals inoculated with vBAC-NS3_435_, vBAC-NS3_480_, vBAC-E_405_/NS3_435_, vBAC-E_402_/NS3_480_ or vBAC-E_402_/NS3_209_/NS3_480_ displayed more severe signs of disease. Almost all of the animals in these groups displayed encephalitis and partial paralysis of the hind limbs (Figure S1). In the groups for which deaths were recorded, 29% of the animals inoculated with vBAC-NS3_435_ died and the mortality rate was even higher (61%) for mice inoculated with vBAC-NS3_480_. These results highlight the importance of the NS3_435_ and NS3_480_ mutations for the acquisition of the viral neurovirulent phenotype. Furthermore, mortality reached 73% in the group of animals inoculated with the double-mutant virus, vBAC-E_405_/NS3_435_, and inoculation with vBAC-E_402_/NS3_480_ and vBAC-E_402_/NS3_209_/NS3_480_ viruses killed 100% of the animals (Figure 4).

Thus, viruses containing the E_402_ and E_405_ mutations alone were no more virulent than vBACDV1. However, when these mutations were combined with the NS3_480_ and NS3_435_ mutations respectively, the resulting viruses, each of which carried two of the mutations found in the neuroadapted derivatives (FGA/NA d1d and FGA/NA P6; Table 1), were neurovirulent.

To assess the ability of the recombinant viruses (vBACDV1, vBAC-E_402_ and vBAC-E_405_) to interact with Neuro 2A cell receptors, binding assays were carried out (Figure S2). No significant difference in binding capacity was observed between these viruses.

**Figure 5:**
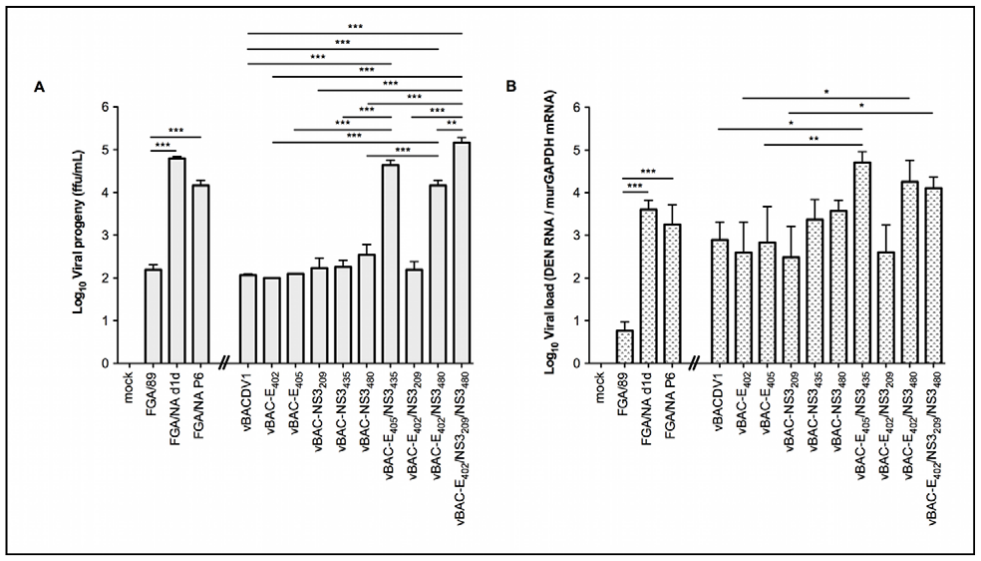
DENV detection at 8 dpi in the brains of mice inoculated with DENV-1 variants. (A) Viral progeny numbers in the mice CNS were determined by titration in C6/36 cells. (B) Viral RNA levels in the mouse CNS were determined by RT-qPCR with normalization against levels of murGAPDH mRNA. Data were log transformed and analyzed by one-way ANOVA followed by Bonferroni's correction for multiple testing and the values presented are the means ± SD of three different experiments. * p<0.05, ** p<0.01 and *** p<0.001. A gap was inserted into the x axis to facilitate data interpretation, by grouping mouse-adapted strains and non mouse-adapted recombinant viruses and their corresponding controls. The significance bars correspond to comparisons between viruses with the mutations at same positions.

We previously showed that viral replication in the brains of mice inoculated with the FGA/89 and FGA/NA P6 strains of DENV-1 peaked nine days after inoculation [52]. To evaluate the replication properties of the recombinant viruses, brains of three animals were collected from each group on the eight day after inoculation, before the onset of signs of disease and death. RT-qPCR analyses and viral titration performed on the brain tissues of animals inoculated with the panel of viruses showed that vBAC-E_405_/NS3_435_, vBAC-E_402_/NS3_480_ and vBAC-E_402_/NS3_209_/NS3_480_ produced the largest numbers of viral progeny and the highest levels of RNA synthesis (Figure 5) in the brain tissues of infected animals, consistent with the high frequency of encephalitis in these animals later in the incubation period (see Figure 4).

**Figure 6:**
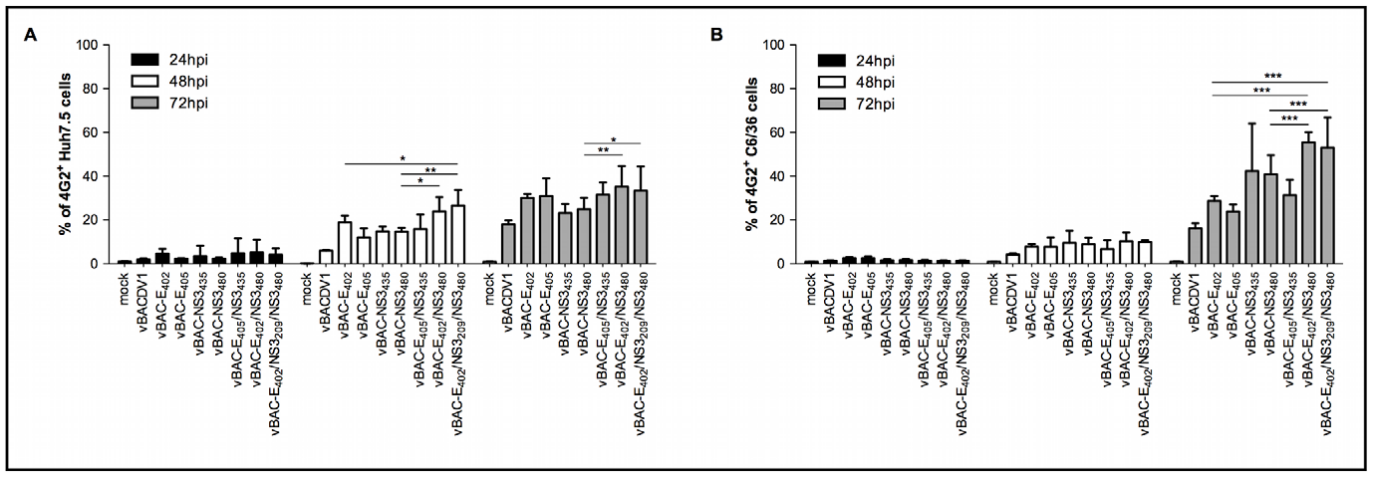
*In vitro* growth kinetics on human and mosquitoes derived cells. (A) Growth kinetics in Huh7.5 cells. (B) Growth kinetics in C6/36 cells. Infected cells were assessed by flow cytometry. Data were analyzed by two-way ANOVA followed by Bonferroni's correction for multiple testing and the values presented are the means ± SD of three different experiments. * p<0.05, ** p<0.01 and *** p<0.001.

We investigated whether the neurovirulent phenotype resulted from an increase in viral fitness by carrying out *in vitro* growth kinetics studies on human and insect derived cells and quantifying protein synthesis. Levels of protein synthesis were significantly higher in Huh7.5 and C6/36 cells infected with vBAC-E_405_/NS3_435_, vBAC-E_402_/NS3_480_ and vBAC-E_402_/NS3_209_/NS3_480_ than in cells infected with vBACDV1 (Figure 6).

**Figure 7:**
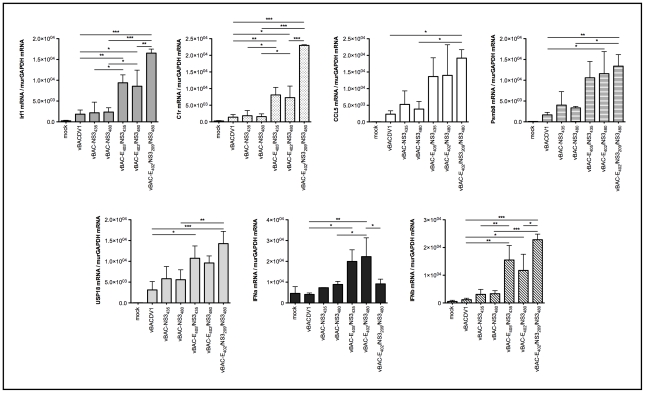
Levels of immunity genes mRNAs in mice brains collected 8 days after infection. The x axis shows DENV-1 variants and the y axis shows the relative levels of mRNAs detected with respect to murGAPDH mRNA. Data were analyzed by one-way ANOVA followed by Bonferroni correction and the values presented are the means ± SD of three different experiments. * p<0.05, ** p<0.01 and *** p<0.001. The significance bars correspond to comparisons between viruses that recover the virulent phenotype.

Results from a previous study ([63] and unpublished results]) revealed that a number of innate immune response genes were differentially expressed in the brains of mice infected with avirulent and neurovirulent strains of DENV-1. Therefore, to analyze the influence of individual mutations on the ability of recombinant viruses to induce innate immunity genes, a subset of genes representing several major pathways [interferon signaling (Irf1 - interferon regulatory factor 1), interferon alpha and beta, antigen presentation (Psmb8 - proteosome subunit beta type 8), protein ubiquitination pathway (Usp18 - ubiquitin specific protease 18), complement system (C1r - component 1, r subcomponent) and chemokine (CCL5 - chemokine ligand 5-C-C motif)] were selected for analyses. RNAs extracted from brain tissues obtained 8 days after infection, were subjected to amplification with specific primers for these genes, and the RT-qPCR signals obtained were normalized with respect to the signal for murGAPDH (Figure 7). Consistent with the virulence (Figure 4) and viral load studies (Figure 5), levels of expression for all of the host genes shown in Figure 7 were significantly higher in animals infected with FGA/NA d1d, FGA/NA P6 (data not shown) or any of the recombinant viruses containing double and triple mutations (vBAC-E_402_/NS3_480_, vBAC-E_405_/NS3_435_ and vBAC-E_402_/NS3_209_/NS3_480_) than in mock-infected or vBACDV1-infected animals.

To discard an eventual mouse to mouse variation due to the outbred nature of the mice used in this study, and confirm the role of the critical residues responsible for increased viral load and pathogenesis, single animals were euthanized at various time points during infection (6, 8 and 10 dpi) and individual mouse CNS and spinal cord tissues were analyzed. Viral RNA synthesis, viral load curves and modulation of innate immune response genes were correlated with disease and death of the animals infected with vBAC-E_402_/NS3_209_/NS3_480_ compared to mock-infected or vBACDV1-infected animals (Figure S3).

We also evaluate the target cells and the damage caused by virus infection in the CNS of these mice, by carrying out histological analyses of brain tissues. Brain tissue collected (10^th^ dpi), from animals infected with neuroadapted (FGA/NA d1d and FGA/NA P6) or recombinant viruses, displayed moderate to severe meningitis. The degree of tissue injury observed (data not shown) was consistent with viral RNA replication, viral load (Figure 5) and the severity of infection as determined by mortality rate (Figure 4).

## Discussion

Several studies have provided support for the hypothesis that viral virulence determinants play a role in dengue pathogenesis and vector transmissibility [71]–[73]. In this study we focused on determining how point mutations, acquired during the adaptation of DENV to mice increase viral fitness *in vitro* and *in vivo*, and exert their effects on mice neuropathogenesis. We used reverse genetics techniques to sample individual mutations found in two independently obtained newborn mouse-adapted isolates of DENV-1 [51]–[52]. Comparisons of the genomes of the parental (FGA/89) and neuroadapted variants of DENV-1 (FGA/NA d1d and FGA/NA P6) suggested that acquired mutations in the genes encoding E and NS3 might be responsible for the neurovirulence of these mouse-adapted strains. To test the role of these mutations in viral fitness and virulence, we created a panel of non-mouse adapted infectious clone-derived viruses with the E mutations (E_402_ Phe to Leu and E_405_ Thr to Ile) and NS3 mutations (NS3_209_ Val to Ile, NS3_435_ Leu to Ser and NS3_480_ Leu to Ser) present separately, in paired or in group of three mutations, as in the empirically adapted isolates. Both of the E mutations studied mapped to the region outside the ectodomain and the three NS3 mutations studied here are located in the helicase region of NS3.

The positions of the mutations detected in the neuroadapted isolates (E_402_ and E_405_ – Figure 2) were not consistent with a change in affinity for the receptor. Indeed, the recombinant viruses carrying these mutations had the same binding affinity for Neuro2A cells as the infectious clone-derived virus. We therefore conclude that the mechanism by which E protein mutations increases virulence involves critical steps occurring after viral attachment (fusion/assembly/release).

Chen and coworkers [74] reported similar results concerning the effect of mutations affecting this domain of E protein on neurovirulence in mice. They used chimeric DENV-4 carrying the C-prM-E genes of DENV-3 to show that a mutation at E_406_ (substitution of a Lys for the WT Glu) increased the neurovirulence of a DENV-4/DENV-3 chimera.

Lin and coworkers [68], using site-directed mutagenesis and functional assays, demonstrate the involvement of the EH1 and EH2 domains of the E protein in DENV assembly and cell entry. Substitutions at positions E_401_ (Met to Pro), E_405_ (Thr to Pro), E_408_ (Gly to Pro) and E_412_ (Met to Pro) in the EH1 domain affected the assembly of DENV VLPs, probably due to interference with prM-E heterodimerization. The authors hypothesized that mutations mapping to the N-terminal EH1 domain affected the association of the stem region with the viral membrane altering curving and bending during the assembly in the ER.

The NS3_209_, mutation, which was co-selected with the NS3_480_ in FGA/NA P6, had no apparent effect on virulence in our studies. The triple mutant recombinant virus (E_402_/NS3_209_/NS3_480_) gave higher viral RNA levels and virus titers in the mouse CNS 8 dpi than the double mutant (E_402_/NS3_480_), but both viruses killed 100% of the animals by days 15 and 16 post infection, respectively.

The recombinant viruses harboring mutations at residues NS3_435_ and NS3_480_, located in the helicase subdomain 2, after motifs V and VI (Figure 3), respectively, displayed an alteration of replicative capacity (*in vitro* and *in vivo*) and were neurovirulent in mice. It has been reported that a substitution at position 249 (Thr to Pro) of the NS3_hel_ in West Nile virus confers a highly virulent phenotype on strains usually only weakly virulent in American crows [75]. This region is involved in RNA binding and ATP hydrolysis and is required to drive the helicase along its nucleic acid substrate [76]. The presence of mutations in these regions may affect the activity of the helicase, increasing replication efficiency, through either a direct effect on helicase activity itself or through interaction with other viral or cellular proteins. Sampath and colleagues [46] carried out a structure-based mutational analysis and proposed an “inchworm” model of DENV NS3 translocation and unwinding activity. They suggested that the pocket next to DENV-2 NS3 Ile_365_ (tip of domain II) would acts as a “helix opener” disrupting hydrogen bonds at the fork. The basic concave face between domains II and III would acts as “the translocator” in this model, by binding dsRNA ahead of the fork. The NS3_480_ mutation maps to this concave face, the NS3_435_ mutation maps to domain III, and both may therefore enhance dsRNA binding and modulate helicase activity.

Grant and coworkers [54] recently described a DENV-2 strain causing lethal infections in immunocompromised AG129 mice. One critical virulence determinant at the NS4B_52_ protein had been identified. By reverse genetics, these authors demonstrated that the replacement of a Leu residue by a Phe residue, at this position, converted a non-virulent strain into a strain causing 80% lethality and increased viremia independently of the host type I interferon response. Physical interaction between NS4B (located in the ER lumen) and NS3 (located on the cytoplasmic face of the ER) is unlikely, but the authors hypothesized that a transient interaction could occur before polyprotein processing, thereby modulating DENV replication and implicating NS3 in this process. They also demonstrated that the NS4B_52_ substitution enhances viral RNA synthesis in mammalian cells but not in C6/36 insect cells.

The non-mouse adapted infectious clone-derived viruses with only the E mutations identified in this study (E_402_ and E_405_) had no higher binding affinity to Neuro2A cells receptor(s) or higher levels of viral RNA synthesis, viral load (*in vitro* and *in vivo*) and neurovirulence in mice than vBACDV1. However, the combination of these mutations with NS3_hel_ mutations (E_405_/NS3_435_, E_402_/NS3_480_ and E_402_/NS3_209_/NS3_480_), altered viral replicative capacity across other tissue (spinal cord) and cell types (Huh7.5 and C6/36 cells) and resulted in a highly neurovirulent phenotype in mice.

The pathological outcome of an infection is determined by the balance between the host response to infection and the ability of the infectious agent to escape from this response and multiply in the host. As part of this dynamic interaction, the host responses to some infections, including DENV infections, may contribute to the pathophysiology of disease. We have shown that high levels of replication of genetically defined DENV result in the upregulation of genes induced by type I IFN (IFN-α/β), consistent with previous data from non human primates [77] and primary cultures of human cells [78].

In a previous study, we investigated the effect of DENV-1 infection on the transcription profile of CNS of mice. The Ube2l6 gene, which encodes an ubiquitin conjugate enzyme, was found to be up regulated in animals infected with the FGA/89 and with a neuroadapted derived strain FGA/NA a5c, with fold changes of 2.59 and 4.73, respectively, eight dpi ([63] and unpublished results). In a recent study based on the use of a high-throughput two hybrid assay, a human cellular protein, with a similar function, UBE2l (an ubiquitine conjugate enzyme), was found to interact with the DENV NS2B, NS4B and NS5 proteins, and siRNA targeting of this gene inhibited DENV replication [79]. As FGA/NA d1d and FGA/NA a5c differ by only three amino-acid substitutions in the E protein, we will investigate further the modulation of the Ube2l6 protein and its interaction with the replication complex during infection with the recombinant viruses generated in this study. Transcript levels for Usp18, which functions as an ubiquitin cycle enzyme, were positively correlated with higher levels of replication in animals infected with the strains vBAC-E_405_/NS3_435_, vBAC-E_402_/NS3_480_ and vBAC-E_402_/NS3_209_/NS3_480_.

We demonstrated here that single mutations in the DENV-1 E protein and NS3_hel_ domain increase viral fitness, both *in vitro* (human and mosquito-derived cells) and *in vivo*, facilitating early virus emergence during mouse infection consistent with a major role in DENV pathogenesis.

In a context of limited knowledge of the molecular basis of dengue pathogenesis, our results could contribute to the establishment of attenuation strains for vaccine development, and provide insights into virus/host interactions and new information about the mechanisms of dengue pathogenesis.

## Supporting Information

Figure S1 **Newborn mice morbidity after i.c. inoculation with DENV-1 variants.** The graphs show the cumulative signs of disease from three independent biological replicates. (A) Comparison of mock, FGA/89 and neurovirulent strains FGA/NA d1d and FGA/NA P6, (B) Comparison of mock, vBACDV1 and single-mutatnt recombinant viruses, (C) Comparison of mock, vBACDV1 and double- and triple-mutant recombinant viruses.
(TIF)

Figure S2 **Assay of the binding of vBACDV1, vBAC-E_402_ and vBAC-E_405_ recombinant viruses to Neuro-2a cells.** Data were analyzed by one-way ANOVA followed by Dunn's multiple comparison test and values are expressed as means ± SD of three different experiments. * p<0.05.
(TIF)

Figure S3 ***In vivo*** **growth kinetics in CNS and spinal cord tissues of individual mice after i.c. inoculation with mock, vBACDV1 and vBAC-E_402_/NS3_209_/NS3_480_.** (A) Viral progeny numbers in the CNS of individual mice were determined by titration in C6/36 cells. (B) Levels of mRNAs of innate immune genes from CNS of individual mice were determined by RT-qPCR with normalization against levels of murGAPDH mRNA. (C) Viral RNA levels in the spinal cord tissue of individual mice were determined by RT-qPCR with normalization against levels of murGAPDH mRNA. #1, #2 and #3 represent each individual animal collected for each respective time point (6, 8 and 10 dpi).
(TIF)

Table S1 **Summary of amino acid sequence differences between vBACDV1 and the GenBank-deposited sequences of BR/90 (used to generate clone pBACDV1), FGA/89 (parental virus used for neuroadaptation), FGA/NA d1d and FGA/NA P6 (neuroadaptated variants from FGA/89).**
(DOC)
