# Supplementary figures and images for: Synergistic Interactions between the NS3hel and E Proteins Contribute to the Virulence of Dengue Virus Type 1
Source: PLoS Negl Trop Dis. 2012 Apr 17;6(4):e1624. doi: 10.1371/journal.pntd.0001624 (PMC3328427; doi:10.1371/journal.pntd.0001624)

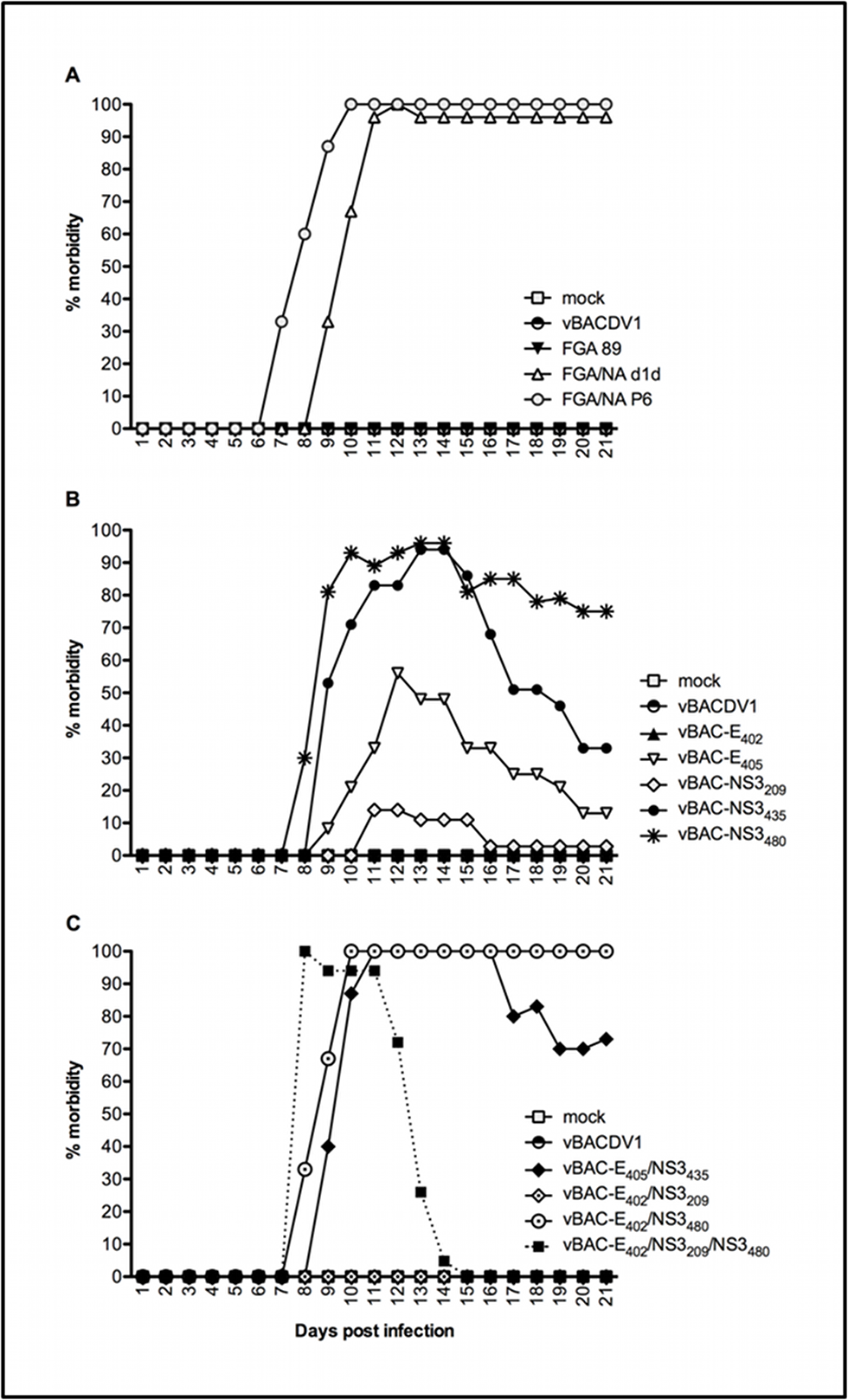

Supplement: Figure S1 — Newborn mice morbidity after i.c. inoculation with DENV-1 variants. The graphs show the cumulative signs of disease from three independent biological replicates. (A) Comparison of mock, FGA/89 and neurovirulent strains FGA/NA d1d and FGA/NA P6, (B) Comparison of mock, vBACDV1 and single-mutatnt recombinant viruses, (C) Comparison of mock, vBACDV1 and double- and triple-mutant recombinant viruses. (TIF) [file pntd.0001624.s001.tif]

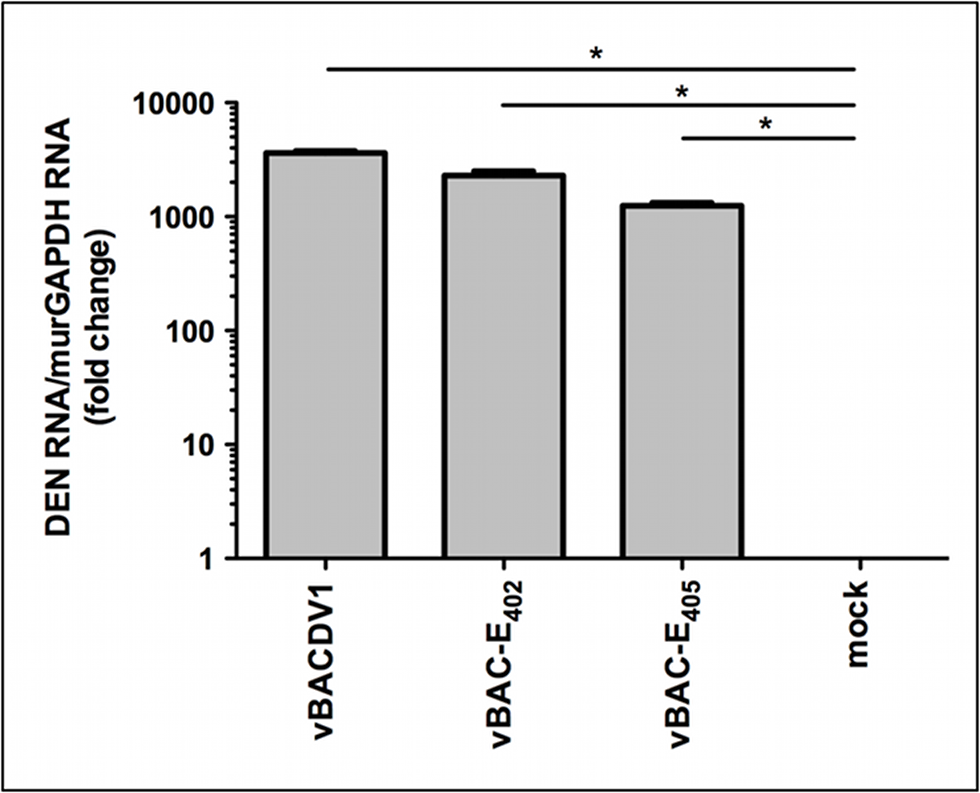

Supplement: Figure S2 — Assay of the binding of vBACDV1, vBAC-E402 and vBAC-E405 recombinant viruses to Neuro-2a cells. Data were analyzed by one-way ANOVA followed by Dunn's multiple comparison test and values are expressed as means ± SD of three different experiments. * p<0.05. (TIF) [file pntd.0001624.s002.tif]

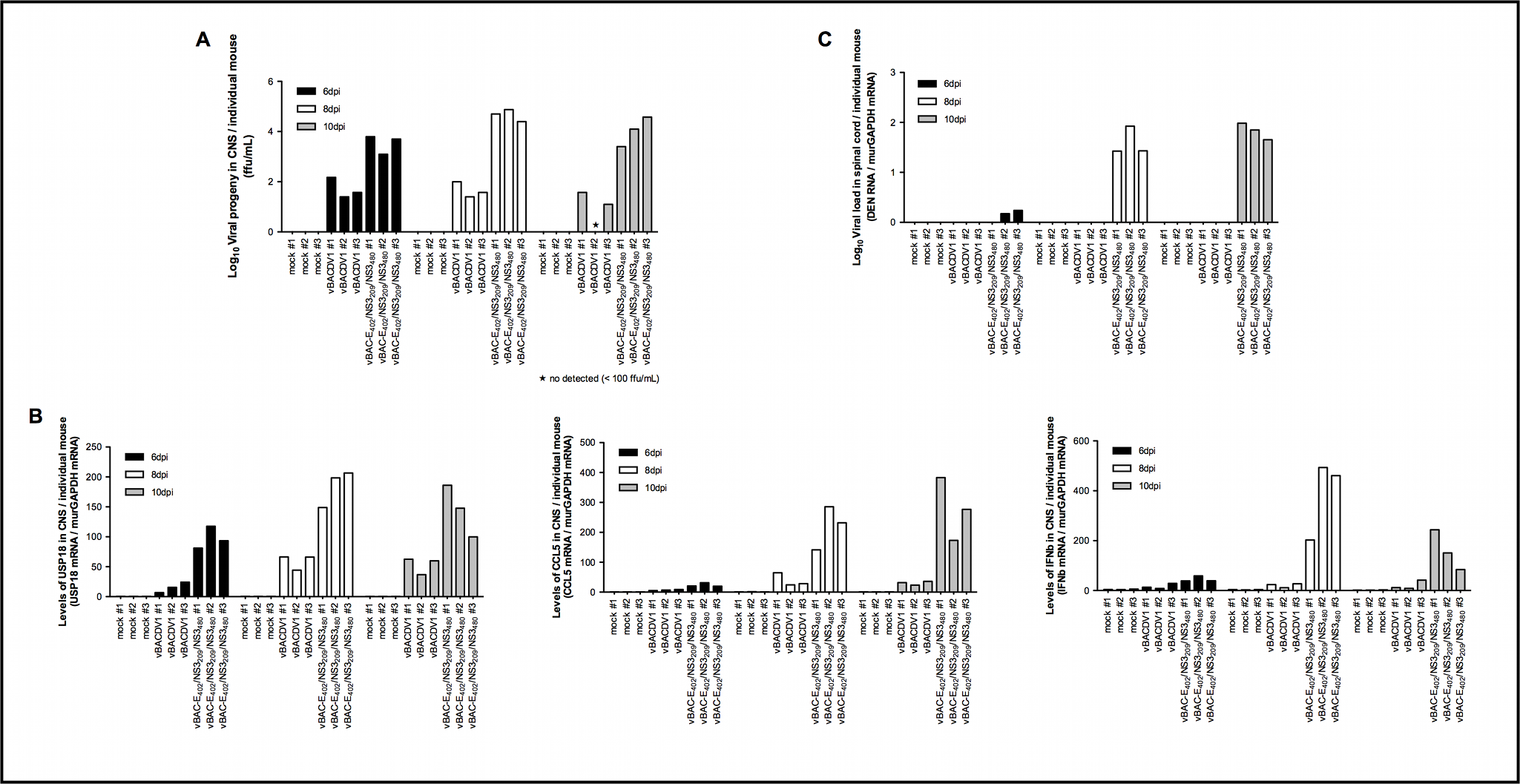

Supplement: Figure S3 — In vivo growth kinetics in CNS and spinal cord tissues of individual mice after i.c. inoculation with mock, vBACDV1 and vBAC-E402/NS3209/NS3480. (A) Viral progeny numbers in the CNS of individual mice were determined by titration in C6/36 cells. (B) Levels of mRNAs of innate immune genes from CNS of individual mice were determined by RT-qPCR with normalization against levels of murGAPDH mRNA. (C) Viral RNA levels in the spinal cord tissue of individual mice were determined by RT-qPCR with normalization against levels of murGAPDH mRNA. #1, #2 and #3 represent each individual animal collected for each respective time point (6, 8 and 10 dpi). (TIF) [file pntd.0001624.s003.tif]
