# Supplementary material for: Synergistic Interactions between the NS3hel and E Proteins Contribute to the Virulence of Dengue Virus Type 1
Source: PLoS Negl Trop Dis. 2012 Apr 17;6(4):e1624. doi: 10.1371/journal.pntd.0001624 (PMC3328427; doi:10.1371/journal.pntd.0001624)
Supplement: Table S1 — Summary of amino acid sequence differences between vBACDV1 and the GenBank-deposited sequences of BR/90 (used to generate clone pBACDV1), FGA/89 (parental virus used for neuroadaptation), FGA/NA d1d and FGA/NA P6 (neuroadaptated variants from FGA/89). (DOC) [file pntd.0001624.s004.doc]

**Table S1.** Summary of amino acid sequence differences between vBACDV1 and the GenBank-deposited sequences of BR/90 (used to generate clone pBACDV1), FGA/89 (parental virus used for neuroadaptation), FGA/NA d1d and FGA/NA P6 (neuroadaptated variants from FGA/89).

| **Coding region** | **Codon position ^a^** | **BR/90 ^b^ and**  **vBACDV1** | **FGA/89 ^c^** | **FGA/NA**  **d1d ^d^** | **FGA/NA**  **P6 ^e^** |
| --- | --- | --- | --- | --- | --- |
| C | 112 | A | V | V | V |
| E | 96 | F | V | V | V |
| E | 180 | A | T | T | T |
| E | 196 | M | M | V | M |
| E | 297 | T | M | M | M |
| E | 365 | V | V | I | V |
| E | 379 | V | I | I | I |
| **E** | **402** | **F** | **F** | **F** | **L** |
| **E** | **405** | **T** | **T** | **I** | **T** |
| E | 473 | T | A | A | A |
| NS1 | 128 | I | T | T | T |
| NS1 | 146 | E | D | D | D |
| NS2A | 67 | K | R | R | R |
| NS2A | 97 | A | T | T | T |
| NS2A | 168 | T | M | M | M |
| **NS3** | **209** | **V** | **V** | **V** | **I** |
| **NS3** | **435** | **L** | **L** | **S** | **L** |
| NS3 | 465 | S | N | N | N |
| **NS3** | **480** | **L** | **L** | **L** | **S** |
| NS4A | 69 | T | M | M | M |
| NS4A | 89 | T | M | M | M |
| NS4B | 24 | Q | H | H | H |
| NS4B | 26 | A | V | V | V |
| NS5 | 114 | V | I | I | I |
| NS5 | 135 | M | I | I | I |
| NS5 | 375 | V | M | M | M |
| NS5 | 399 | T | I | I | I |
| NS5 | 784 | I | V | V | V |

^a^ Position of codon change within the individual protein-encoding region.

^b^ AF226685.2.

^c^ Parental strain from which the neuroadapted strains were derived (AF226686.2).

^d^ Neuroadapted strain (AF226686.1).

^e^ Neuroadapted strain (EF122231.1).
